# Geschichte der Volksseuchen nach und mit den Berichten der Zeitgenossen, mit Berücksichtigung der Thierseuchen

**Published:** 1897-03

**Authors:** 


### Reviews of Books.

---

**Geschichte der Volksseuchen nach und mit den Berichten der Zeitgenossen, mit Berücksichtigung der Thierseuchen.** Von Dr. B. M. Lersch. Pp. 455. Berlin: S. Karger. London: Williams and Norgate. 1896.

This is a useful work of reference, giving in a brief and succinct form the annals of the various pestilences which have been recorded from the earliest times. Some of these diseases, as the author says, have disappeared and some have become more active at the present day; but both for the historian and the physician it is desirable to have at hand concise descriptions of the prevalence of epidemics in their chronological order. In the short space of 450 pages are crowded a vast mass of facts, gathered both from contemporary and modern writers. Thus the recent works of Jessopp and Gasquet are laid under contribution for the outlines of the Black Death in England and elsewhere, which the author identifies with the Oriental bubonic plague, and to which he devotes some forty pages. Numerous references to the authorities and literature of the subjects are given, and on the whole the work impresses us highly. The printing is excellent; but the index is poor.

